# Systems Metabolic Engineering of Industrial Microorganisms

**DOI:** 10.3390/microorganisms11040926

**Published:** 2023-04-03

**Authors:** Xueqin Lv, Yu Wang, Boyang Ji, Xiao-Jun Ji

**Affiliations:** 1 Key Laboratory of Carbohydrate Chemistry and Biotechnology, Ministry of Education, Jiangnan University, Wuxi 214122, China; 2 Science Center for Future Foods, Jiangnan University, Wuxi 214122, China; 3 Key Laboratory of Engineering Biology for Low-Carbon Manufacturing, Tianjin Institute of Industrial Biotechnology, Chinese Academy of Sciences, Tianjin 300308, China; 4 BioInnovation Institute, Ole Maaløes Vej 3, 2200 Copenhagen, Denmark; 5 State Key Laboratory of Materials-Oriented Chemical Engineering, College of Biotechnology and Pharmaceutical Engineering, Nanjing Tech University, Nanjing 211816, China

The green and sustainable production of chemicals, materials, fuels, food, and pharmaceuticals has become a key solution to the global energy and environmental crisis [1]. Microbial fermentation provides an efficient and reliable method for renewable production [2]. Therefore, scientists are committed to developing industrial microbial cell factories to produce bio-based products in a green and efficient manner to meet the industrial production needs [3]. Several bio-based chemicals, including 1,3-propanediol [4], succinic acid [5], and 1,4-butanediol [6], have been produced on an industrial scale through systems metabolic engineering.

However, it is still challenging to develop microbial cell factories that meet industrialization and commercialization requirements [7]. The complexity of cell metabolism and its related regulatory networks is one of the major challenges in developing precisely regulated microbial cell factories [8]. Furthermore, the production of exogenous and unnatural molecules requires the expression of heterologous and novel enzymes and pathways [9]. These unnatural elements often cause metabolic competition, imbalance, and inhibition in host strains [10]. All of these require systematic exploration of the cellular metabolism and physiological properties of microorganisms [11]. Additionally, efficient and precise genome editing, gene regulation tools, and various components with specific functions, such as catalysis and transport, are indispensable for developing industrial microorganisms [12]. Further modifications and optimizations are required for higher production levels (titer, yield, and productivity) and the stable performance of scaled-up fermentation using industrial microorganisms [13].

In this Special Issue, different strategies for the combinatorial engineering of industrial microorganisms, the development of regulatory tools, and the exploration of production processes were reported. Liu et al. [14] summarized the progress of research on the regulation of riboflavin production in *Bacillus subtilis* and reviewed different strategies for enhancing riboflavin production through metabolic engineering. Satta et al. [15] provided an overview of the latest innovative biotechnology and systems metabolic engineering strategies used for cyanobacteria. Diao et al. [16] screened and overexpressed acetoin-resistance genes in *Escherichia coli* and strengthened the (*R*)-acetoin synthesis pathway, thereby enabling the engineered *E. coli* strain GXASR-49RSF to produce 81.62 g/L of (*R*)-acetoin, exhibiting its great potential for (*R*)-acetoin production. Li et al. [17] developed an *E. coli* SRP-4 strain with high colanic acid production at 30 °C by increasing the precursor supply and alleviating the regulator of capsule synthesis phosphorylation system. In a 3 L bioreactor, 24.99 g/L of low-molecular-weight colanic acid was produced, the highest titer reported so far. Kim et al. [18] assessed the safety of a newly isolated *B. subtilis* strain IDCC1101, and performed genome sequencing to investigate the functional genes involved in secondary metabolites, virulence, antibiotic resistance, and mobile elements. *B. subtilis* IDCC1101 was proven safe for industrial applications. Yang et al. [19] overexpressed 14 genes related to cell polarization in *Saccharomyces cerevisiae*. Overexpression of BUD1, CDC42, AXL1, and BUD10 increased the activity of surface-displayed α-amylases, whereas the overexpression of BUD1, BUD3, BUD4, BUD7, and BUD10 enhanced the activity of secreted α-amylases. In *Pseudomonas aeruginosa*, Konopacki et al. [20] characterized the hydrodynamic parameters such as mixing time, power consumption, and mass transfer, in a 2 L bioreactor and found that rhamnolipid production was most efficient with moderate oxygen mass transfer and low-intensity mixing. Tian et al. [21] analyzed the effects of bisphenol A on *Rhodococcus equi* DSSKP-R-001 in terms of its growth and metabolism, gene expression pattern, and antitoxin mechanism. The results indicated that cytochrome P450 monooxygenases and multicopper oxidases play key roles in bisphenol A degradation. In *Moesziomyces antarcticus*, Nascimento et al. [22] reported the production of mannose erythritol lipids using oils produced by microalgae at 1.78 ± 0.04 g/L/day. Wu et al. [23] integrated enzyme kinetics and proteomics data to build the first genome-scale enzyme constraint model of *B. subtilis* (ecBSU1) using the ECMpy workflow. They used ecBSU1 to identify potential target genes that enhance the production of commodity chemicals, demonstrating that ecBSU1 can guide the rational design of microbial cell factories.

In a production process study, Cui et al. [24] proposed a novel “microbial nanofluid,” which contained petroleum hydrocarbon-degrading bacteria (PHDB) and SiO_2_ nanoparticles to enhance residual oil recovery. The results showed that residual oil recovery increased by 30.1% compared with water flooding at the optimum composite concentration of “microbial nanofluid”, which contained PHDB (7.0%, *v*/*v*) and nano-SiO_2_ (100 mg/L). Al-Khairy et al. [25] described the bioengineered production of different polymers, including polylactic acid and polyhydroxyalkanoates (PHA and PHB), and provided information on the metabolic pathways for PHA and PHB production in bacteria. They highlighted the bioengineering approaches, new developments, and challenges in plastic bioproduction and biodegradation.

A systems-level understanding of cellular metabolism, including gene expression, enzyme activity, regulation and metabolite interactions, is critical to better engineering industrial microorganisms in the future. We believe that this series of papers can provide references for systems biology-guided cell factory engineering, further promote the integration of systems biology and metabolic engineering approaches, and provide theoretical guidance for the construction and optimization of microbial cell factories for producing bio-based chemicals, fuels, and materials.
